# Control in the absence of choice: A qualitative study on decision-making about gastrostomy in people with amyotrophic lateral sclerosis, caregivers, and healthcare professionals

**DOI:** 10.1371/journal.pone.0290508

**Published:** 2023-09-08

**Authors:** Remko M. van Eenennaam, Neele Rave, Willeke J. Kruithof, Esther T. Kruitwagen-van Reenen, Leonard H. van den Berg, Johanna A. Visser-Meily, Anita Beelen

**Affiliations:** 1 Department of Rehabilitation, Physical Therapy Science and Sports, UMC Utrecht Brain Center, University Medical Center Utrecht, Utrecht, The Netherlands; 2 Center of Excellence for Rehabilitation Medicine, UMC Utrecht Brain Center, University Medical Center Utrecht and De Hoogstraat Rehabilitation, Utrecht, The Netherlands; 3 Department of Neurology, UMC Utrecht Brain Center, University Medical Center Utrecht, Utrecht, The Netherlands; Ege University, Faculty of Medicine, TURKEY

## Abstract

**Background:**

Gastrostomy is recommended in amyotrophic lateral sclerosis for long-term nutritional support, however, people with amyotrophic lateral sclerosis and healthcare professionals perceive decision-making as complex.

**Method:**

To explore their perspectives on decision-making regarding gastrostomy, we used semi-structured interviews with people with amyotrophic lateral sclerosis, who had made a decision, and their caregivers; healthcare professionals were interviewed separately. Interviews were transcribed and analyzed thematically.

**Results:**

In 14 cases, 13 people with amyotrophic lateral sclerosis and 12 caregivers were interviewed; and in 10 of these cases, 5 healthcare professionals. Participants described decision-making on gastrostomy as a continuous process of weighing (future) clinical need against their values and beliefs in coming to a decision to accept or reject gastrostomy, or to postpone decision-making, while being supported by loved ones and healthcare professionals. Participants described gastrostomy as inevitable, but retained agency through control over the timing of decision-making. They said physical necessity, experiences of loss and identity, and expectations about gastrostomy placement were important factors in decision-making. Decision-making was described as a family affair, with caregivers supporting patient choice. healthcare professionals supported people with amyotrophic lateral sclerosis during the decision-making process and respected their autonomy and values. People with amyotrophic lateral sclerosis stressed the importance of adequate information on the procedure and the benefits.

**Conclusion:**

People with amyotrophic lateral sclerosis feel in control of decision-making on gastrostomy if they are able to make their own choice at their own pace, supported by loved ones and healthcare professionals. Person-centered decision-making on gastrostomy requires early information exchange and repeated discussions with people with amyotrophic lateral sclerosis and their caregivers, incorporating their values and respecting patient choice.

## Introduction

Amyotrophic lateral sclerosis (ALS) is characterized by progressive loss of function and a median survival of three years [1]. Increasing difficulty with swallowing (i.e. dysphagia) and chewing, and reduced upper limb function can lead to weight loss and malnutrition which are independent prognostic factors for survival in ALS [2, 3]. Furthermore, dysphagia can result in aspiration, choking, recurring chest infections, and increasingly prolonged and effortful meals that negatively influence quality of life and cause distress to people with ALS and their caregivers [4]. Gastrostomy, either via percutaneous endoscopic gastrostomy (PEG) or percutaneous radiological gastrostomy (PRG), is recommended to provide long-term nutritional support and reduce the risk of aspiration [5–7]. However, benefits in promoting survival, weight, or quality of life are less apparent compared to, for example, non-invasive ventilation [3, 8–12]. Furthermore, healthcare professionals (HCPs) struggle with the lack of evidence-based cut-off values or criteria for indicators (e.g. weight loss, respiratory function, and dysphagia) to support optimal timing of placement [5, 12–14]. Finally, disease course and rate of disease progression vary per person with ALS, making patient choice and values an important aspect of decision-making on gastrostomy. However, HCPs and people with ALS [4, 15, 16] experience this decision-making process as complex and difficult.

HCPs may favor a proactive approach to symptom management in ALS [17] and can experience a lack of patient readiness to make a decision as a barrier to (timely) decision-making on gastrostomy [13, 14], especially because delayed placement increases the risk of complications with little nutritional benefit, and can make placement impossible due to deteriorating health [5, 18]. However, recommendations for earlier placement [3, 12, 19] sit uneasily with people with ALS for whom the impact of gastrostomy goes beyond clinical and nutritional factors [20]. To them, accepting or foregoing gastrostomy is one of many difficult decisions on their journey and they may prefer to postpone decision-making [21, 22]. Reasons include reluctance to give up eating, anxiety about the procedure, and desire to remain in control [4, 15, 16, 23]. People with ALS are supported by caregivers during the course of their disease; however, considerations on perceived caregiver burden can also influence decision-making [14, 15, 24, 25]. Furthermore, during the disease course loss of speech and hand function – for electronic or text-to-speech communication – may impair their ability to communicate and around half of people with ALS develop cognitive and behavioral impairments with one in eight fulfilling the criteria for frontotemporal dementia [1], both of which may complicate the decision-making process [26]. Greater insight into the viewpoint of all primary stakeholders (people with ALS, caregivers, and HCPs) is necessary in order to fully capture the dynamics and complexities of the decision-making process in each particular case.

In this study, therefore, we explored the experiences of people with ALS, their caregivers, and their HCPs with the decision-making process on gastrostomy. Greater insight into the perspectives and experiences of the primary stakeholders will improve support and allow tailoring of information and decision-making to the needs of people with ALS and caregivers, while promoting patient choice.

## Methods

### Standard protocol approvals, registrations, and patient consent

The study protocol was submitted to the Medical Ethical Committee of the university medical center (UMC) Utrecht (19-583/C) who deemed it exempt from review as the Dutch Medical Research Involving Human Subjects Act was not applicable. Participation was voluntary and written consent was obtained after informing patients and caregivers about the study. If patients were unable to provide written consent due to impaired hand or upper limb function, verbal consent was registered by their caregiver on the informed consent form.

### Setting

In the Netherlands, people diagnosed with ALS are referred for care to one of 35 multidisciplinary ALS care teams where ALS care is part of (rehabilitation) palliative care. ALS care teams are coordinated by a rehabilitation physician. Four ALS care teams were involved in the recruitment for this study: UMC Utrecht, Utrecht (where the majority of participants were recruited); Tolbrug Rehabilitation Center, Den Bosch; Rehabilitation Center Klimmendaal, Arnhem; Rijndam Rehabilitation Center, Erasmus Medical Center, Rotterdam.

### Participants

#### Patients and caregivers

Patients were eligible for inclusion if they had a diagnosis of ALS, progressive muscular atrophy (PMA) or primary lateral sclerosis (PLS), an indication for gastrostomy, had made a decision to either accept or decline gastrostomy, and, in the former case, gastrostomy had been placed. Caregivers were eligible to participate if they had been involved in the decision-making process. Patients with cognitive impairments or impaired or absent speech were eligible for inclusion as long as a caregiver was willing to participate in the interview. Patients and their caregivers were recruited by rehabilitation physicians at the four participating ALS care teams in the Netherlands and by one neurology nurse specialist (UMC Utrecht). Patients who expressed interest and their caregivers were sent an information leaflet on the study and contacted by one of the researchers (RvE, NR) to inform them about the study. After informed consent to participate had been obtained, written consent was provided; if patients were unable to provide written consent due to impaired hand or upper limb function, verbal consent was registered by their caregiver on the informed consent form. After written consent was received, a date, time and interview mode (i.e. face-to-face, video-call or telephone) convenient to the participants were agreed. The interviewers were not known to the participants prior to contacting them for this study.

#### HCPs

Patients were asked to nominate their HCP who had greatest involvement in decision-making on gastrostomy; they were also invited to participate in a separate interview. RvE was known to two of the HCPs (EKvR, WK), because they work at the same institution (UMC Utrecht) and are part of the research team.

### Data collection

#### Patients and caregivers

Semi-structured interviews with detailed probes were conducted by two researchers (RvE, NR) not involved in the care of patients. RvE has been trained to conduct qualitative research and NR has been coached and supervised in conducting interviews and qualitative analysis by RvE. Both RvE and NR were supported by a senior researcher with extensive experience in qualitative research (AB). The interviews were directed by an interview guide formulated on the basis of a literature review (RvE, AB; S1 File). Patients with impaired or absent speech were offered the option to first respond via e-mail to the interview questions. These answers were used to prepare for the interview. Taking patient preferences into account, the interview was conducted and recorded via telephone or video-consultation.

At the start of the interview participant characteristics (gender, age, level of education, diagnosis, decision on gastrostomy (yes or no), method of gastrostomy insertion (PEG, PRG, other, or none), and relationship of caregiver to patient) were registered. During the interview, patients and their caregivers were invited to elaborate on their experiences with the decision-making process regarding gastrostomy: when and how this was discussed, their reasons for accepting or rejecting gastrostomy, the role of HCPs and significant others, and their satisfaction with their decision and the decision-making process. If a decision had been made to accept gastrostomy, they were also asked about the advantages and disadvantages of living with a feeding tube. At the end of the interview or via e-mail, patients and caregivers were asked a few sensitive questions, without the other being present. The patient was asked about the roles of perceived caregiver burden and of significant others in the decision-making process. Caregivers were asked about the burden of mealtimes before and after the placement of the feeding tube, and whether cognitive changes in the patient may have affected the decision-making process. Participants were offered a transcript of the interview to allow corrections and additions (member check).

#### HCPs

Separate, semi-structured interviews with detailed probes were conducted with HCPs by RvE. The interviews focused specifically on the decision-making process on gastrostomy of the patient who had nominated the HCP and were directed by an interview guide on the basis of a literature review (RvE, AB; S2 File). At the start of the interview, HCP characteristics were registered (age, position, years of experience with ALS). During the interview, HCPs were asked about when (i.e. at what point in the disease process) and how gastrostomy was discussed with the patient, how the decision-making process proceeded, the dynamics of decision-making between patient-caregiver and HCP, and satisfaction with the decision-making process.

### Data analysis

#### Patients and caregivers

Interviews were transcribed verbatim, anonymized, and analyzed by two researchers (RvE, NR) using an inductive approach. The process of data collection and analysis was iterative, proceeding simultaneously to provide the opportunity for important emerging topics to be incorporated into subsequent interviews. Inclusion proceeded until data saturation was reached, i.e. when no new themes emerged during the last three interviews [27]. First, transcripts were read to become familiar with the narrative. Second, the texts were broken down into fragments based on their content and coded independently by two researchers (RvE, NR) in NVIVO 12 (NVivo Qualitative Data Analysis Software; v. 12.6) using open coding [28]. Resulting codes and discrepancies were compared and discussed to enhance credibility of the results and minimize interpretation bias. Third, after every 4-5 interviews, existing codes were evaluated by the research team (RvE, NR, AB, WK) and, where necessary, recoded. Fourth, codes were sorted and categorized into overarching themes and subthemes using thematic analysis [29]. A descriptive summary of each theme was written and quotes were linked to the themes by one researcher (RvE) to express the essence of the content; themes were discussed by the research team (RvE, NR, AB, WK, EKR).

#### HCPs

In a similar procedure, the HCP interviews were transcribed verbatim, anonymized, and analyzed by RvE, as described above.

## Results

### Participants

In 14 cases, a total of 14 interviews were carried out with thirteen people with ALS and twelve caregivers, between June 2020 and August 2021 (Table 1). In 11 cases, dyads were interviewed together; in case 2, the daughter assisted her father in communication without participating in the interview; in case 11, the person with ALS was too tired to participate due to rapid disease progression; case 14 lived alone in a nursing home. In twelve cases the diagnosis was ALS, one PLS, and one PMA; twelve cases had had gastrostomy at the time of interview. Interviews took between 21 and 68 minutes. Data saturation was reached after ten people with ALS and nine caregivers had been interviewed. All of the participants wanted and received transcripts of their interviews; they provided no comments or feedback on the transcripts.

**Table 1:** Description of cases.

Case	Participant	Sex	Age	Education	Diagnosis	Type of gastrostomy	Mode of communication (person with ALS)
C1	Person with ALS 1	Female	75	High	PLS	PRG	Written, speech computer, making sounds, & non-verbal
	Partner 1	Male	74	High			
C2	Person with ALS 2	Male	65	High	ALS	PRG	Verbal (sometimes difficult to understand)*
	Daughter 2	Female	34	High			
C3	Person with ALS 3	Female	69	High	ALS	PEG	Written, speech computer, making sounds, & non-verbal
	Daughter 3	Female	31	High			
C4	Person with ALS 4	Female	52	Intermediate	ALS	PRG	Written, speech computer, making sounds, & non-verbal
	Partner 4	Male	60	Intermediate			
C5	Person with ALS 5	Male	75	Intermediate	PMA	None	Verbal
	Partner 5	Female	71	High			
C6	Person with ALS 6	Male	72	High	ALS	PRG	Verbal
	Partner 6	Female	69	Intermediate			
C7	Person with ALS 7	Female	60	High	ALS	PRG	Written & making sounds
	Partner 7	Male	60	High			
C8	Person with ALS 8	Male	62	Intermediate	PLS/ALS **	PRG	Written & verbal (unintelligible, caregiver translates)
	Partner 8	Female	64	High			
C9	Person with ALS 9	Female	63	Intermediate	ALS	PEG	Written & verbal
	Partner 9	Male	64	Intermediate			
C10	Person with ALS 10	Male	46	High	ALS	PRG	Written, verbal (unintelligible, caregiver translates), & non-verbal
	Partner 10	Female	40	High			
C11	Person with ALS 11	Female	69	High	ALS	PRG	None ***
	Partner 11	Male	74	High			
C12	Person with ALS 12	Male	62	High	ALS	PRG	Verbal
	Sister 12	Female	65	High			
C13	Person with ALS 13	Female	60	Intermediate	ALS	PRG	Written, speech computer, making sounds, & nonverbal
	Partner 13	Male	60	Intermediate			
C14	Person with ALS 14	Male	78	High	ALS	None	Verbal

* Daughter assisted her father in communication without participating in the interview

** Person with ALS was originally diagnosed with PLS which later converted to ALS

*** Person with ALS was too tired to participate.

Additionally, four rehabilitation physicians and one neurology nurse specialist were interviewed about the decision-making process in 10 of 14 cases. HCPs’ age ranged from 34 to 58 years and their experience with ALS ranged from 1 to 15 years. Three rehabilitation physicians were not interviewed (cases 6, 7, and 11): one declined to participate, one was on maternity leave, and one did not respond. In one case, no HCP was nominated (case 2) because the person with ALS decided on gastrostomy while he was in the hospital for trial participation.

### Themes of the decision-making process

**Fig 1:**
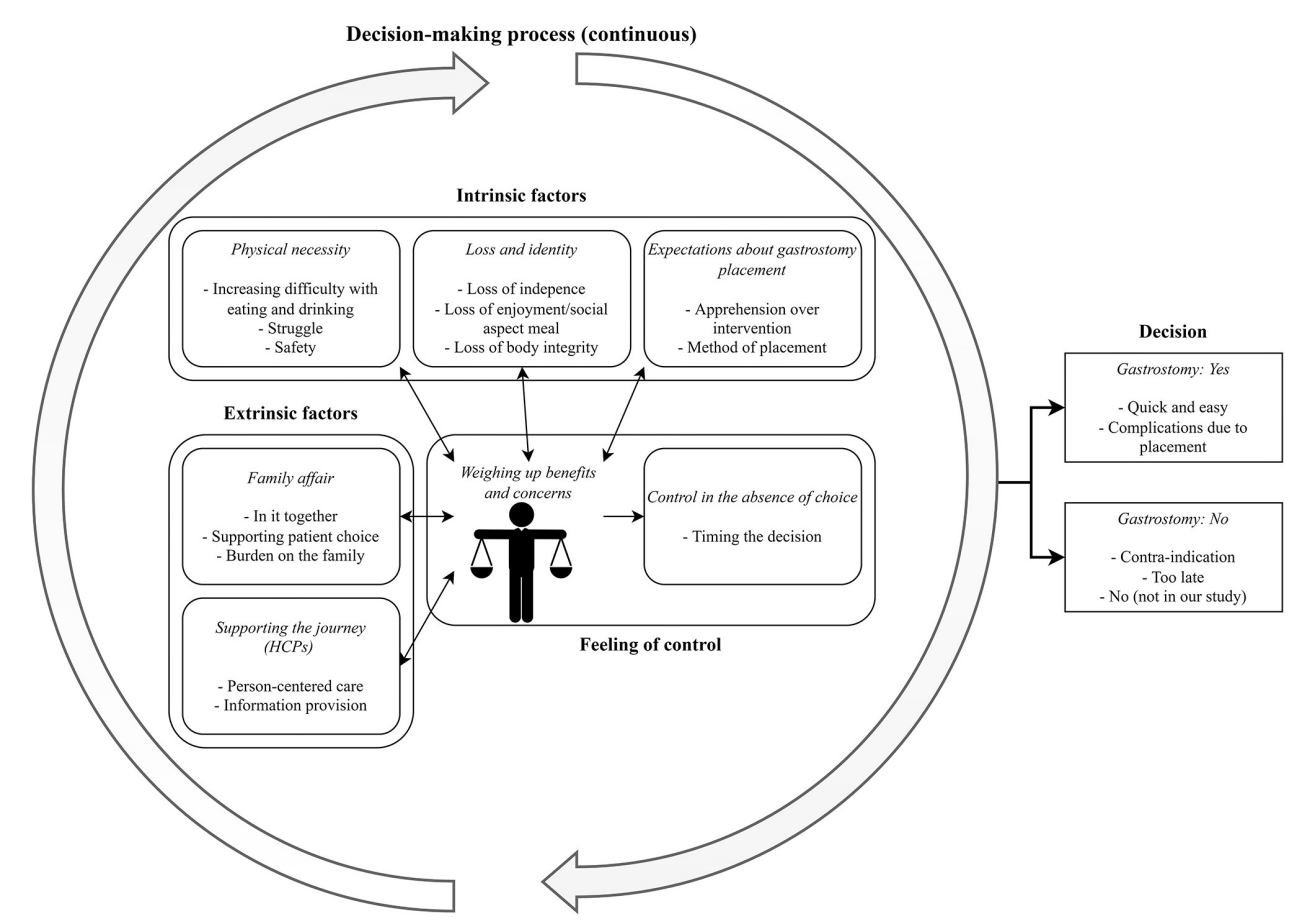
Overarching themes on the experiences of people with ALS, caregivers, and healthcare professionals with decision-making about gastrostomy in amyotrophic lateral sclerosis.

Seven, closely interrelated themes emerged from the interviews; these are graphically presented in Fig 1. Rather than making a decision at a particular moment, decision-making on gastrostomy was described as a continuous process, with people with ALS weighing up the (future) clinical need for gastrostomy against their values, beliefs and expectations, while being supported by significant others and HCPs. People with ALS and their caregivers explained that the weight of these factors shifts over time as the disease progresses. The increasing difficulty with eating and drinking confronts people with ALS with the ongoing loss, thus threatening their identity and forcing them to accept and adapt to change. After deliberating, people with ALS decide to accept or to reject gastrostomy, or to postpone decision-making, on the whole with the support of significant others and HCPs. Finally, despite experiencing an absence of choice when confronted with the progression of their disease, people with ALS explained they experienced control over the decision-making process by controlling the timing and because of the support from their significant others and HCPs.

### Feeling of control

*Weighing up benefits and concerns*. Some participants explained that for them their concerns weighed more heavily than their clinical needs and the possible benefits; consequently, they declined gastrostomy or delayed decision-making for as long as possible (quotes 1, H1 in Table 2). Emotions associated with loss and identity played an important role, gastrostomy often being viewed as a threat to their independence that would reduce them from being a person to being a patient (quotes 1, H1, 2), but also, to a lesser degree, apprehension over the intervention (see theme ‘expectations about gastrostomy placement’). However, over time the weight of these factors would begin to shift as clinical needs began to outweigh their concerns; as the disease progressed, it became harder and harder to ignore the impact of increasing difficulty with eating and drinking, and deterioration in health (quote 2, 3, 4). In the end, they felt they could not delay the decision any longer and were forced to make the ‘difficult but necessary’ decision to accept gastrostomy (quote 2). Others said physical concerns and their quality of life trumped their emotions and concerns, and they accepted gastrostomy soon after the indication was discussed (quote 5). Information on possible benefits could make their decision easier (see theme ‘supporting the journey’).

**Table 2:** Quotes on ‘feeling of control’ in gastrostomy decision-making.

Themes and subthemes	Quotes
*Weighing up benefits and concerns*	
	Person with ALS 8: “Dismissive, because of the dependency it would inevitably create. … It was a pragmatic decision, which we postponed until it was no longer responsible to do so.” **Quote 1. Person with ALS 8**
	HCP 2: “One of the things was that [he] categorically did not want to be dependent on others. That thought held him back for a long time, also in relation to the decision as to whether or not to opt for tube feeding.” **Quote H1. HCP 2 on person with ALS 8**
	Person with ALS 4: “I wanted to know more [when gastrostomy was first discussed], but wasn’t ready for it yet. I could still eat. … After a year [I accepted gastrostomy.]… Difficult but necessary… Eating was becoming difficult and I was losing weight fast… I see it as yet another step backwards. … I also wanted to continue to eat independently, no matter how difficult it was.” **Quote 2. Person with ALS 4**
	Partner 4: “You’re both perfectly aware of what’s coming and that, sooner or later, you’re literally not going to have a choice. … Eating got so difficult at one point … choking a lot, taking in very little food, that [she] also started realising it was inevitable. She really didn’t have a choice.” **Quote 3. Partner 4**
	Person with ALS 10: ‘Not in the middle of 2015 [when gastrostomy was discussed] but early in 2020 did I decide to accept a feeding tube … because I started to choke more often and swallowing became more difficult. And I started to lose weight.’ **Quote 4. Person with ALS 10**
	Person with ALS 6: “Then the rehabilitation doctor suggested we start with tube feeding. And I actually accepted that straight away. … The fear of losing weight was much greater than looking into the possible consequences of tube feeding.” **Quote 5. Person with ALS 6**
*Control in the absence of choice*	
Absence of choice	Partner 8: “There really was no way around opting for tube feeding if we wanted to try and maintain the weight. Person with ALS 8: [says something unintelligible] Partner 8: “No, that’s right, you didn’t have a choice. That’s right. You reach a point where all that’s left is what you can still do, rather than about what you want to do.” **Quote 6. Person with ALS 8 & partner 8**
	Partner 11: “But of course you’ve started looking into the disease a bit more at this stage, so it didn’t exactly come as a huge shock. You know beforehand that it’s going to happen at some point. She was eating all day, then the choice isn’t all that difficult.” **Quote 7. Partner 11**
	Person with ALS 1: “Eating and drinking became increasingly more difficult. No [not a hard decision], consuming food normally turned into a downright disaster. … It was simply a fait accompli.” **Quote 8. Person with ALS 1**
Timing the decision	Person with ALS 10: “Not a hard decision, I knew it was coming. … I’m in control of things myself. So I decide whether or not I want to do something. …” Partner 10: “He also knew that [tube feeding was inevitable] and you were sort of delaying that.” Person with ALS 10: “Yes.” **Quote 9. Person with ALS 10 & Partner 10**
	HCP 3: “She did have control and, as her weight had stabilised, there was no great urgency to get it done. But the fact that her swallowing function was also continuing to decline meant she knew it needed to be done. … She managed to retain her dignity, in the sense that she liked to stay in control of things herself.” **Quote H2. HCP 3 on person with ALS 13**
	Person with ALS 2: “I’m completely open to as many adjustments and aids as possible, as long as they help make life worth living. … I decided to indicate that I wanted to make that choice. … You’re much more dependent on others without aids.” **Quote 10. Person with ALS 2**
	Partner 7: “We especially didn’t want to end up in a situation where we no longer had a choice. And you can really only make things easier for yourself if you preventively can stay one step ahead of the inevitable.” **Quote 11. Partner 7**
	HCP 4: “This is an extraordinary patient who, apparently, can accept things very easily and actually turn them into something positive. … Normally discussing both a PEG and non-invasive ventilation is quite stressful for people, as this once again indicates a huge step and a machine. … He took that incredibly well.” **Quote H3. HCP 4 on person with ALS 12**
	HCP 2: “Right from the beginning he had also said, ’yes, at some point it’s going to be necessary, but I don’t want it yet’. So I think with him it’s always been more of a no-not-yet scenario, rather than a definitive no.” **Quote H4. HCP 2 on person with ALS 8**
	Person with ALS 4: “Ultimately made the decision myself. … I thought that initial meeting, more than a year earlier, was too soon and no one was difficult about this. …” Partner 4: “[She] decides what happens to her and no one else. She was able to make that choice herself. I’m sure she felt that was very important.” **Quote 12. Person with ALS 4 & partner 4**
	Person with ALS 3: “More or less forced into it because I continued losing weight. … I no longer had any interest in life as an ALS patient during the time I had to decide on tube feeding. I was severely depressed. …” Daughter 3: “We, the family, more or less pushed [the decision] through together with the healthcare professionals. …” Person with ALS 3: “Hardly a choice… it ended up being forced. … But [in retrospect] that PEG isn’t as bad as you think.” **Quote 13. Person with ALS 3 & daughter 3**
	HCP 1: “I have, on occasion thought, ‘how is he cognitive?’ … I can’t always be sure, especially as he doesn’t always completely answer your questions. … But it simply doesn’t have any further consequences and [his wife] dismisses that too.” **Quote H5. HCP 1 on person with ALS 5**

*Control in the absence of choice*. People with ALS and caregiver interviews revealed a fundamental paradox at the root of decision-making on gastrostomy: making a decision in the absence of choice, i.e. feeling “forced” by the progressive nature of ALS. During the course of their disease, most people with ALS became convinced, they said, that deteriorating physical function, worsening nutritional status, and weight loss, would make gastrostomy – now or at some point in the future – necessary and unavoidable (quotes 3, 6, 7, 9). In fact, many of them accepted early on in the disease that a feeding tube might someday be necessary and thus it did not come as a surprise to them when gastrostomy indication was discussed (quote 7). In some cases, this absence of choice made the decision to accept gastrostomy easier (quotes 7, 8), but others felt that it threatened their independence, making them postpone the inevitable (quotes 1, 9).

Despite the feeling of not having a choice, people with ALS reported that they retained a feeling of control and agency over the decision-making process and the final decision, through the timing of their decision (quotes 9, H2). Some decided on early placement before there was a clear indication (quotes 10, 11). Others made a quick, pragmatic decision when the indication was discussed with them (quotes 5, 7, 8, 11, H3). Finally, there were those who initially declined or postponed decision-making as long as possible to preserve their independence (quotes 1, H1, 9). HCPs explained that rejection of a feeding tube is often a strategy of people with ALS to maintain their independence and a temporary rather than a categorical rejection: a ‘no, not yet’ rather than a ‘no, never’ (quote H4).

All involved – people with ALS, caregivers, and HCPs – emphasized the importance of the person with ALS retaining control and facilitating that control (quotes 12, 28, 29, H8, H10). Only one of the people with ALS said they were pressured by significant others or their HCPs to make a decision in favor of or against gastrostomy. The one person who did feel pressured said she felt forced to accept a feeding tube by her family and HCPs after she became depressed and stopped eating (quote 13). None of the caregivers reported changes in personality or cognitive functioning of the person with ALS that impacted on his/her decision-making capacity; nor did the HCPs. In one case the HCP explained that the possibility of co-occurrence of cognitive deficits and communication impairments can sometimes make it difficult to assess the decision-making capacity of that person with ALS; however, this was not the case for this person with ALS according to the HCP (quote H5).

### Intrinsic factors

*Physical necessity*. People with ALS and their caregivers described how, over time, mealtimes became increasingly difficult with eating and drinking becoming a challenge (quotes 14, 15 in Table 3). They explained that they struggled to sustain the weight and adequate nutritional status of the person with ALS; this would take an increasing amount of time and energy (quotes 14, 16); loss of appetite might also play a role for some participants (quote 17). As swallowing became more difficult, safety would become an increasing point of concern (quotes 3, 18).

**Table 3:** Quotes on ‘intrinsic factors’ in gastrostomy decision-making.

Themes and subthemes	Quotes
*Physical necessity*	
Increasing difficulty with eating and drinking	Person with ALS 1: “Eating normally became a true disaster…because it simply fell out of the mouth. …” Partner 1: “Those lovely meal moments turned into a confrontational drama. … [And] the weight loss that really needed to stop. … That’s obviously life-threatening.” **Quote 14. Person with ALS 1 & partner 1**
	Person with ALS 6: “Eating more just wasn’t possible. … Your very fine motor skills are gone from your hands.” **Quote 15. Person with ALS 6**
Struggle	Person with ALS 9: “I had to work incredibly hard throughout the day to ingest enough food to maintain my weight … and drinking became trickier too … My tongue can’t do an awful lot anymore.” **Quote 16. Person with ALS 9**
	Person with ALS 6: “My appetite had all but disappeared because both my smell and taste had gone. So that inevitably results in you eating a great deal less.” **Quote 17. Person with ALS 6**
Safety	Person with ALS 14: “I found eating [increasingly] more difficult. … It seemed to go wrong every time, pieces would get stuck in your trachea and then you’d end up being short of breath.” **Quote 18. Person with ALS 14**
*Loss and identity*	
Loss of autonomy	Partner 8: “I think it’s mainly about the fact that this will definitely make [him] a patient. That was the biggest stumbling block. … [He] is a very autonomous human being. … Being dependent on others is a very sensitive subject to him and he would have liked to have avoided that at all cost.” **Quote 19. Partner 8**
	Person with ALS 4: “[I] wasn’t ready for it yet. … I found it incredibly difficult to give something up yet again. …” Partner 4: “You keep on having to give up a little bit of your quality of life, which then, putting it bluntly, brings you one step closer to death every time. … That’s obviously really confrontational.” **Quote 20. Person with ALS 4 & partner 4**
	Person with ALS 7: “It wasn’t a difficult decision. Although it *was* one which had a great deal of impact, as it was yet another step backwards. Mentally it’s incredibly hard to keep giving in.” **Quote 21. Person with ALS 7**
Loss of enjoyment/ social aspect meal	Person with ALS 1: “No more taste experiences. I really miss the joy of eating together. …” Partner 1: “Of course it’s not particularly social.” **Quote 22. Person with ALS 1 & partner 1**
Loss of body integrity	Person with ALS 3: “Difficult decision, invalidating, unnatural. … I hated it. There’s a hole in the body and something which is always visible, so it instantly feels like a disability.” **Quote 23. Person with ALS 3**
*Expectations about gastrostomy placement*	
Apprehension over intervention	Daughter 3: “She was very anxious about it and dreading the pain and then there’s obviously pain afterwards. That’s horrible.” **Quote 24. Daughter 3**
	Person with ALS 4: “I [was] dreading being admitted to hospital, because I can no longer do anything independently.” Partner 4: “[She] is almost completely paralysed, she can’t speak, and then when you end up in the care of others and you can’t express exactly what you want or what you’re looking for… that’s obviously really, really difficult. They weren’t able to get her out of bed properly, use a hoist, and what more.” **Quote 25. Person with ALS 4 & partner 4**
Method of placement	Person with ALS 9: “It’s much better to have a PEG tube placed when you’re feeling fit, then having a PRG tube placed in a worse condition, as this needs to be replaced every four months and the balloon can burst. I really don’t like the idea of that.” **Quote 26. Person with ALS 9**

*Loss and identity*. As their disease progressed, people with ALS reported being confronted by loss, related to mealtimes, eating, and the idea of a feeding tube which threatened their values and identity. Emotions associated with increasing dependency and becoming a patient were particularly strong for some (quotes 1, H1, 19). It was difficult to give up eating, and gastrostomy was viewed as yet another step in the progression of their disease (quotes 20, 21). The loss of enjoying taste and the social aspect of meals was also remarked upon by a number of participants (quote 22). Loss of body integrity might also be relevant. One person described the idea of a feeding tube as a violation of the integrity of her body (quote 23), and another participant said he viewed being dependent on all kinds of machines to continue living as unnatural (quote 48).

*Expectations about gastrostomy placement*. Some participants described being apprehensive about the placement of a feeding tube, due to fear of pain (quote 24), and because they felt helpless, due to their inability to communicate and control their own body (quote 25). This apprehension could be exacerbated or ameliorated by the information provided by HCPs (see theme ‘supporting the journey’). A few people with ALS said they would prefer a PEG rather than a PRG and, therefore, decided on early, timely placement (quote 26).

### Extrinsic factors

*Family affair*. When discussing the decision-making process about gastrostomy, people with ALS and their significant others described it as a family affair and emphasized that they were in it together (quotes 27, H6 in Table 4). People with ALS said they felt supported during decision-making by their family and loved ones, who emphasized the importance of respecting the choice and autonomy of the person with ALS (quotes 12, 28,29). However, as mealtimes became increasingly difficult, caregivers did report increasing feelings of helplessness and worry about the person with ALS’ health and safety, which could turn mealtimes into a source of tension for both of them (quotes 29, 30) and increase the caregivers’ burden of care (quote 31, 32). In these cases, some caregivers reported discussing gastrostomy more frequently (quote 33). Also, one couple described the traumatic impact of their young child seeing his father choking and the added strain on the mother (quote 32). However, caregivers did not complain about their burden of care (quote 29, 31), and people with ALS said their decision had not been determined by concerns about caregivers’ burden of care (quote 29).

**Table 4:** Quotes on ‘extrinsic factors’ in gastrostomy decision-making.

Themes and subthemes	Quotes
*Family affair*	
In it together	Person with ALS 13: “In consultation with my partner. … We work together to figure out the best solutions. …” Partner13: “We decided that together.” **Quote 27. Person with ALS 13 & partner 13**
	HCP 2: “[Partner] supported him unconditionally. Was also a pragmatic person, solution-focused … so they certainly always did that together.” **Quote H6. HCP 2 on person with ALS 8**
Supporting patient choice	Partner 10: “[He] likes to be in control. Of course we’ve discussed this together, but it’s [his] body and it’s also [his] decision.” **Quote 28. Partner 10**
	Person with ALS 9: “My husband has always supported me and I don’t think this is too much for him … I’ve not allowed myself to be influenced by my environment. They don’t know what it’s like to be me and what eating and drinking is like for me.” Partner 9: “Absolutely not, I do it with love. But of course you notice that it results in a certain amount of tension in yourself too.” **Quote 29. Person with ALS 9 & partner 9**
Burden on the family	Person with ALS 8: “I wasn’t [eating] enough, my partner tried to encourage that. Eating pretty much turned into an obsession. …” Partner 8: “You’re naturally worried about it. You can already see someone’s losing weight and that aversion to food starts to build up. So I really tried to push as much as I could.” **Quote 30. Person with ALS 8 & partner 8**
	Partner 15: “Now I’m feeding all day. So that burden has now shifted to me, although it’s obviously no burden to me whatsoever, but it *is* my responsibility.” **Quote 31. Partner 15**
	Person with ALS 10: “Also when children are around … which can obviously leave them with some traumatic experiences.” Partner 10: “[He] has choked badly enough on a few occasions that he literally couldn’t breathe for a significant amount of time. … We have a seven year old son.” Person with ALS 10: “He used to crawl behind the sofa.” Partner 10: “… He certainly wasn’t the best eater either and that sometimes demanded some attention too. So the whole process of cooking, eating, clearing up etc could take a couple of hours in total.” **Quote 32. Person with ALS 10 & partner 10**
	Partner 4: “It’s a process and it takes a very long time. I’d been saying that for months and at a certain point she realised there’s no other way.” **Quote 33. Partner 4**
*Supporting the journey (HCPs)*	
Person-centered care	HCP 2: “I think that’s actually one of the great things about our profession, that you can get so close to someone – make it so personal, think along with someone like this, empathise – and watch that person go through the process of arriving at a decision like that.” **Quote H7. HCP 2 on person with ALS 14**
	HCP 2: “I really do strongly [feel] that tube feeding should be any individual’s own choice: are you or are you not going to do it. As long as you’re clearly informed of what it will result in, all the pros and cons. … Who am I in this? Why would I push for tube feeding?” **Quote H8. HCP 2 on person with ALS 8**
	Person with ALS 8: “Brochures and personal conversations. The latter were particularly helpful. [Healthcare professionals were] informative and pleasant, so much so that we could ultimately draw our own conclusions. The [specialist nurse] didn’t apply any pressure. We were able to grow towards that decision as a result of the step-by-step information and all the conversations over time.” **Quote 34. Person with ALS 8**
	HCP 2: “We discussed it several times. … The dietician, for example, mentioned it a few times. … So it’s always been a topic of conversation … in a nice way. And then came that informative conversation with the [specialist nurse], during which he was given an explanation and that certainly gave him something to think about.” **Quote H9. HCP 2 on person with ALS 8**
	HCP 3: “It was very much up to her to identify that ‘yes, now’ moment. … This is a patient who hugely values self-management and who wants to wait for as long as possible, despite being made aware of all the pros and cons.” **Quote H10. HCP 3 on person with ALS 13**
	Partner 4: “That’s confrontational. … These can sometimes be introduced to the conversation at a very early stage.” **Quote 35. Partner 4**
Information provision	HCP 2: “There are also no definite cut-off values for [optimal timing of placement] … This gentleman had swallowing problems, things were by no means optimal and he was losing weight. And yes, then you do have more of a sliding scale, the indication was certainly there before, but it wasn’t a case of “imminent death” either.” **Quote H11. HCP 2 on person with ALS 8**
	Person with ALS 9: “The speech therapist told me I needed a great deal of energy for both talking and eating and that this [feeding tube] would mean I didn’t need to fight quite so hard. The PEG nurse reassured me about the procedure. … The fact that I can just use it for water or put medication in it too.” **Quote 36. Person with ALS 9**
	Daughter 3: “We also received an explanation from the specialist nurse. … And I thought she really clearly explained what it’s like for the patient. … Of course that it’s quite nerve-racking, but also explaining about the placement and what happens afterwards. … I thought that was all very pleasant. …” Person with ALS 3: “She [nurse specialist] was better than the doctor.” **Quote 37. Person with ALS 3 & daughter 3**
	Person with ALS 13: “Nice conversation [with the rehabilitation doctor]. …” Partner 13: “I don’t think you have a great deal of choice about whether or not to do it. … And perhaps the decision-making is made just that little bit easier during these conversations, because you’re being so effectively informed about everything.” **Quote 38. Person with ALS 13 en partner 13**
	Person with ALS 6: “It was a personal choice, but definitely hugely influenced by what the rehabilitation doctor said … that this is the only option if you want to be able to maintain your weight.” **Quote 39. Person with ALS 6**
	Person with ALS 12: “I have to admit to being a little worried about that. Because you do hear some stories about people choking and them almost having to be turned upside down to get it back up again.” **Quote 40. Person with ALS 12**
	Person with ALS 2: “A fellow patient was given a PEG tube. She didn’t think the procedure was too bad. … The decision wasn’t difficult, because I already knew two patients who were being tube fed.” **Quote 41. Person with ALS 2**
	Person with ALS 12: “So [in addition to the tube feeding] all I need to drink is red wine and eat Tony’s Chocolonely [chocolate]. … I only need to eat things I like and I’ll still maintain my weight.” **Quote 42. Person with ALS 12**
	Daughter 3: “Of course inserting such a PEG tube is really just a routine procedure … The nurses talked about it like it was just something to quickly get done. I know it doesn’t take a huge amount of effort, but having a tube inserted into your stomach is no joke.” **Quote 43. Daughter 3**
	Person with ALS 6: “I would have liked … more information about the consequences … and I feel I received little or no information about that.” **Quote 44. Person with ALS 6**

*Supporting the journey (HCPs)*. HCPs described person-centered care aimed at supporting the person with ALS on their journey while respecting their autonomy, values, and dignity (quotes H7, H8); this was echoed by people with ALS and caregivers (quotes 12, 34). Their role during the decision-making process, HCPs explained, was to discuss when gastrostomy was indicated and for what reasons, to make sure the person with ALS was fully informed about the pros and cons, to keep the decision-making process alive by repeatedly discussing the topic as long as a final decision had not been made, while supporting the autonomy of the person with ALS, and respecting their choice and values (quotes H8, H9, H10). Nevertheless, sometimes people with ALS and caregivers felt the topic was raised too early (quote 35).

When coming to an indication, HCPs explained that the timing was relative and dependent on multiple indicators (quote H11). Discussing gastrostomy with people with ALS and their caregivers was seen as a multidisciplinary effort. In addition to the rehabilitation physician, many specialists were involved including dieticians, speech therapists, and specialist nurses (quote H9, 36). People with ALS and caregivers explained that this multidisciplinary approach increased their satisfaction with the decision-making process because of the complementary input provided by HCPs (quotes 34, 36, 37, 38). Information about the feeding tube provided by HCPs could help them reach a decision. Some of the possible benefits were stabilization of weight (quote 39), reduced risk of suffocation (quote 40), reduced energy loss due to effortful meals, and easier intake of medication and fluids (quote 36). Experiences of others with ALS (quote 41), information about still being able to eat as well as tube feed (quotes 42), and reassurance about the intervention (quote 36) were also mentioned as helpful for people with ALS and caregivers in making a decision. However, sometimes participants said HCPs downplayed gastrostomy placement as a minor surgical procedure while not taking sufficient account of their worries (quote 43). Careful explanation of the procedure that is sensitive to the fears and emotions of people with ALS and caregivers can reassure them about the procedure (quote 37). In retrospect, some people with ALS and caregivers also said they would have wanted more information about possible complications and drawbacks of placement and tube feeding (quote 44).

### Decision to accept or reject gastrostomy

Faced with the perceived inevitability of gastrostomy due to the progressive nature of their disease, in our study, all but two people with ALS decided they wanted and received a feeding tube. Once the decision to accept a feeding tube was made, people with ALS and caregivers were happy that the follow-up process was quick and easy, with prompt placement (quote 45 in Table 5). Unfortunately, for a few people with ALS placement caused pain (quotes 24, 46) or emotional distress due to their inability to communicate, which could be further compounded by nursing personnel, lacking experience with ALS (quote 25).

**Table 5:** Quotes on the ‘decision’ to accept or reject gastrostomy.

Themes and subthemes	Quotes
Quick and easy	Person with ALS 7: “We didn’t take a lot of time to decide, so I ended with a feeding tube within a month of my decision.” **Quote 45. Person with ALS 7**
Complications	Person with ALS 2: “[I experienced] an awful lot of pain during the initial week after placement.” **Quote 46. Person with ALS 2**
Contra-indication	Person with ALS 5: “It was 45 [% success versus] 55% [no success]. Well, that’s not a risk I’m willing to take.” **Quote 47. Person with ALS 5**
Too late	Person with ALS 14: “So I said ’I don’t want to do that, I don’t want to live like a plant’. … I didn’t really want anything at the time, I just wanted to go naturally. …. But I did eventually come to the realisation that being tube fed doesn’t mean you’re a plant. … [But the rehabilitation doctor] thought I was already too weak … they no longer thought that was a good idea. … I honestly believe it was simply my own fault.” **Quote 48. Person with ALS 14**
	HCP 1: “Of course you’re glad she didn’t end up with pneumonia and that the procedure went well. That there weren’t a multitude of complications, because that’s obviously the last thing we would have wanted. That was clearly all a downside of waiting. And those [risks] obviously increased for her.” **Quote H12. HCP1 on person with ALS 4**

Of the two people with ALS who did not accept a feeding tube, one explained he did not consider gastrostomy a viable option because of fear of serious complications related to his medical history (quote 47). The other person recounted resolutely rejecting a feeding tube time and again until, very late in the disease course, he changed his mind, but then it was too late because his physician told him he had become too weak (quote 48). None of the participants expressed a principled rejection of gastrostomy.

Although some people with ALS experienced complications from the placement, or strong emotions surrounding loss, none of them expressed regret about their decision or the timing of these decisions; this included the person who felt forced by her family (quote 13) and the one who was too late (quote 48). HCPs also expressed their satisfaction with the decision-making process – regardless of what the person with ALS decided – as long as they had been well-informed and had made the decision based on their own values (quotes H8, H10). In the case of one person who had postponed the decision for a long time, the HCP said she was happy that postponement had not caused any complications (quote H12).

## Discussion

In this study, we show that decision-making on gastrostomy is a complex and continuous process during which people with ALS weigh (future) clinical needs against their values and preferences. They often describe gastrostomy – at some point during their disease – as inevitable, due to the progressive nature of ALS. Nevertheless, despite what they described as an absence of choice, they said they felt in control of decision-making because they were supported by HCPs and loved ones to make their own decision in their own time.

### Feeling of control

On their journey, people with ALS are confronted by a relentless, progressive loss of function and loss of control over their body, that threatens not only their independence, but also their identity and autonomy as a person [30, 31]. In response, they try to retain and regain control over their lives by exerting control over their healthcare, deciding when and how to engage with healthcare services, what aids to accept and when [32]. Regaining control helps promote a feeling of self-worth and personal integrity [31]. This and other qualitative studies [4, 15, 16, 33] show different approaches by people with ALS towards decision-making on gastrostomy ranging from (early) acceptance, postponement, to refusal, based on their individual consideration of physical necessity versus their values and preferences, and expectations about gastrostomy placement. Some may take control and try to get ahead of their disease by choosing proactive, early placement before there is a clear indication; others accept placement soon after the indication is discussed with them [15, 33]. They view gastrostomy as an aid rather than a threat to their independence and quality of life, or as a necessary solution to their increasingly difficult, stressful, and time-consuming struggle with eating and drinking [16, 33]. But even more crucial is their conviction that gastrostomy – now or at some point in the future – would become inevitable [15]. Greenaway et al. suggest [15] that ‘those who felt they had no choice but to accept an intervention’ did not feel in control. However, similar to our findings people with ALS and their caregivers in a recent study by Paynter et al. [34] described a ‘window of opportunity’ in which they still experienced some control before the disease had progressed too much; especially where decision-making about (early) gastrostomy placement was concerned. People with ALS in our study said they felt in control because it was their own decision which they were allowed to make in their own time.

Besides early acceptance, postponement of decision-making and initial refusal may be strategies aimed at protecting independence and retaining control over their lives and healthcare [15], with other factors like reluctance to give up oral feeding, fear of the procedure, a desire to preserve their body integrity, etc. also playing an important role [4, 15, 33]. However, this and other studies [16, 33] show that people with ALS who initially postponed or declined may also come to view gastrostomy as inevitable and end up accepting placement when physical necessity increases and begins to outweigh their feelings of loss, threat to their identity, and concerns over placement. It is, therefore, important to discuss the topic of gastrostomy at regular intervals because even initial and repeated rejections may turn out to be a “no, not yet” rather than “no, never”. In a recent study by Paynter et al. [34] people with ALS and their caregivers also described this ‘window of opportunity’ during which they still experienced some semblance of choice and control over decision-making. Moreover, Paynter et al. [34] suggest that a feeling of control may be what differentiates decision-making about (early) gastrostomy placement from other decisions in ALS which their participants described as not being a decision because there was no choice. We would suggest that most people with ALS – despite coming to view gastrostomy as inevitable at some point during their disease – will feel in control of the decision-making process when they are able to make their own choice, in their own time, and supported by their HCPs and loved ones.

### Supporting the journey (HCPs)

The interrelatedness of patient choice and timing underscores the complex nature that characterizes decision-making on gastrostomy – and other healthcare decisions – in ALS [30, 31]. HCPs can struggle with the lack of evidence about optimal timing of gastrostomy placement and a lack of patient readiness regarding decision-making [13, 14]. Hogden et al. [17] describe a multi-stage model that can support HCPs in engaging people with ALS and their caregivers in patient-centered decision-making. The role of the HCP is to support patient choice and control over decision-making by exploring their preferences and values (participant engagement), establish choices and (optimal) timing of each choice ensuring they have ‘sufficient resources to make informed choices’ (option information), and support them in deciding between ‘proceeding with a symptom management option, and deferring their decision to a later time or choosing to do nothing’ (option deliberation) [17]. Our study shows that presenting the option to accept or decline, or postpone decision-making as valid choices, while explaining advantages and drawbacks of each option, allows people with ALS to make their own, informed decision in their own time while feeling supported by their HCPs, and increasing their satisfaction with and feeling of control over the decision-making process.

Hogden et al. [17] also point out a number of barriers to successful decision-making. Prolonged deliberation because people with ALS delay or initially refuse gastrostomy may cause tension with HCPs’ desire to maximize health outcomes, as was also shown in our survey of rehabilitation physicians in the Netherlands [13]. Late gastrostomy placement is associated with a higher risk of complications, or placement may no longer be feasible due to deteriorating health, as was the case with one person in our study who changed his mind after having repeatedly declined gastrostomy [5, 18]. However, feeling pressured by HCPs to consider and accept gastrostomy can cause people with ALS and their caregivers distress, and damage the relationship with the patient [15]. Patient choice is especially important because the clinical benefits of gastrostomy on survival, weight, or quality of life are less clear-cut and measurable compared to NIV [3, 8–12] and conservative management should be considered a valid option [35]. Accepting well-informed patient choice that could result in an increased risk of complications, or mean that placement is no longer feasible, can be difficult for HCPs, but respects patient autonomy and their values.

During the course of their disease around half of people with ALS develop cognitive impairments, and about one in eight frontotemporal dementia [1], which may impair decision-making capacity [17, 26, 36]. However, only a small minority of people with ALS may actually be incapable of giving consent to treatment [37]; moderate cognitive and behavioral impairment may not impact decision-making on gastrostomy or NIV [38]. We did not succeed in including cognitively impaired people with ALS in our study; more research on the impact of cognitive deficits on healthcare decision-making is needed [26].

Some participants in our study said they felt insufficiently informed about possible complications due to gastrostomy placement. Only when HCPs explain possible benefits, but also potential drawbacks and complications – even if this may cause them to postpone or refuse – will people with ALS, together with their caregivers, be able to make an informed decision [17]. Our study also showed that when gastrostomy placement is described as a minor surgical procedure, people with ALS – who may be largely helpless without the ability to move their arms and legs, and incapable of communication – may feel that not sufficient account is taken of their worries; this runs the risk of increasing their anxiety, whereas careful explanation sensitive to their fears can be reassuring [39]. HCPs walk a difficult tightrope between explaining clinical benefits versus drawbacks, exploring and accommodating values and preferences of people with ALS, and respecting patient choice. Stories of other people with ALS that reflect both the risks and benefits of the different choices might be valuable in helping people with ALS make better informed decisions as long as these are reliable and properly contextualized [33].

### Family affair

ALS has been described as a family illness with loved ones providing emotional support and taking on many aspects of care [40]. This can be a significant burden on caregivers, spouses especially, as the disease progresses [41]. Increasingly difficult mealtimes and food preparation, especially when young children are involved, increase caregiver burden and distress, as well as their worries over the health and safety of their partner [4, 33]. However, tube feeding may come with its own burden on caregivers [42]. Acknowledging and discussing these topics provides an avenue for HCPs to contextualize and discuss patients’ values and decision-making on gastrostomy. People with ALS dislike feeling they are a burden [25]; they may refuse gastrostomy or other interventions, which they perceive to be life-prolonging, in order to not extend the burden of care on loved ones, or accept these – against their own preferences – because their family wants them to carry on living [15, 24, 33]. In our study, people with ALS said caregiver burden did not play a role in their decision to accept or decline gastrostomy. Except for one person who was depressed, none of our participants felt forced or pressured to make a decision against their own preferences. Rather, caregivers emphasized that their partner was not a burden and that it was their decision. As a result, people with ALS in our study did not describe themselves as a burden and said they engaged in collaborative decision-making together with their partners. This underscores the importance of involving loved ones during every stage to facilitate patient-centered decision-making [17].

### Generalizability

Our study and others [4, 15, 16, 33] show the complex and value-laden nature of decision-making on gastrostomy. Similar to ALS, concerns about the impact on social life, body integrity, uncertainty and anxiety about the procedure, and caregiver burden also cause decisional conflict in other diseases [43]. Patients with other progressive diseases (e.g. multiple sclerosis) also emphasized the absence of choice where gastrostomy was concerned [44]. Studies have also shown the importance of the role of HCPs and information provision; HCPs’ poor communication, blasé attitude towards gastrostomy and placement, lack of or inappropriate information, and a paternalistic attitude have resulted in patients and caregivers feeling dissatisfied or excluded from the decision-making process [39, 44]. Our study shows that multidisciplinary ALS care can make it easier to establish a positive relationship with one or more HCPs, and provides information from different, but complementary specialties, helping them to make better, informed decisions. Furthermore, we show that person-centered, multidisciplinary ALS care with HCPs who treat the patient as a person by exploring their values and preferences, respecting their choice, and supporting them during decision-making, reinforces people’s autonomy, and makes them feel in control of and satisfied with the decision-making process.

### Strengths and limitations

An important strength of this study is that all primary stakeholders – i.e. people with ALS, caregiver, and HCP nominated by the person with ALS – were interviewed. The triangulation of multiple viewpoints enhances the credibility of our findings. Another strength of our study is that we included people with ALS with impaired or absent speech, which is often the case by the time gastrostomy becomes relevant. This was made possible through a flexible approach similar to that proposed by Howard et al. (2021) [45]. We provided people with ALS with the opportunity to respond to the questions by e-mail preceding the actual interview. Then they and their caregiver were interviewed together. The opportunity to respond via e-mail also helps to overcome the risk that the views expressed represent those of the caregiver more than of the person with ALS. In the case of absent speech, people with ALS regularly interjected to correct or add to caregiver responses using communication aids, making sounds, or non-verbal communication; cues which were used by the interviewers, where necessary, to ask follow-up questions. Finally, both participants were asked questions on more sensitive topics about caregiver burden and impaired cognition in private (this could also be via e-mail). Another important strength is the qualitative study design which allows us to capture the complexity and individual nature of decision-making on gastrostomy in ALS.

One limitation is that, except for case 11, our study did not include people with ALS with a very fast disease progression. A rapid progression may make it hard to adapt to loss and make a decision in time, before the reality of their disease has overtaken them. But for others, rapid progression can make it easier to accept gastrostomy, because there is no time to delay.

## Conclusion

**Fig 2:**
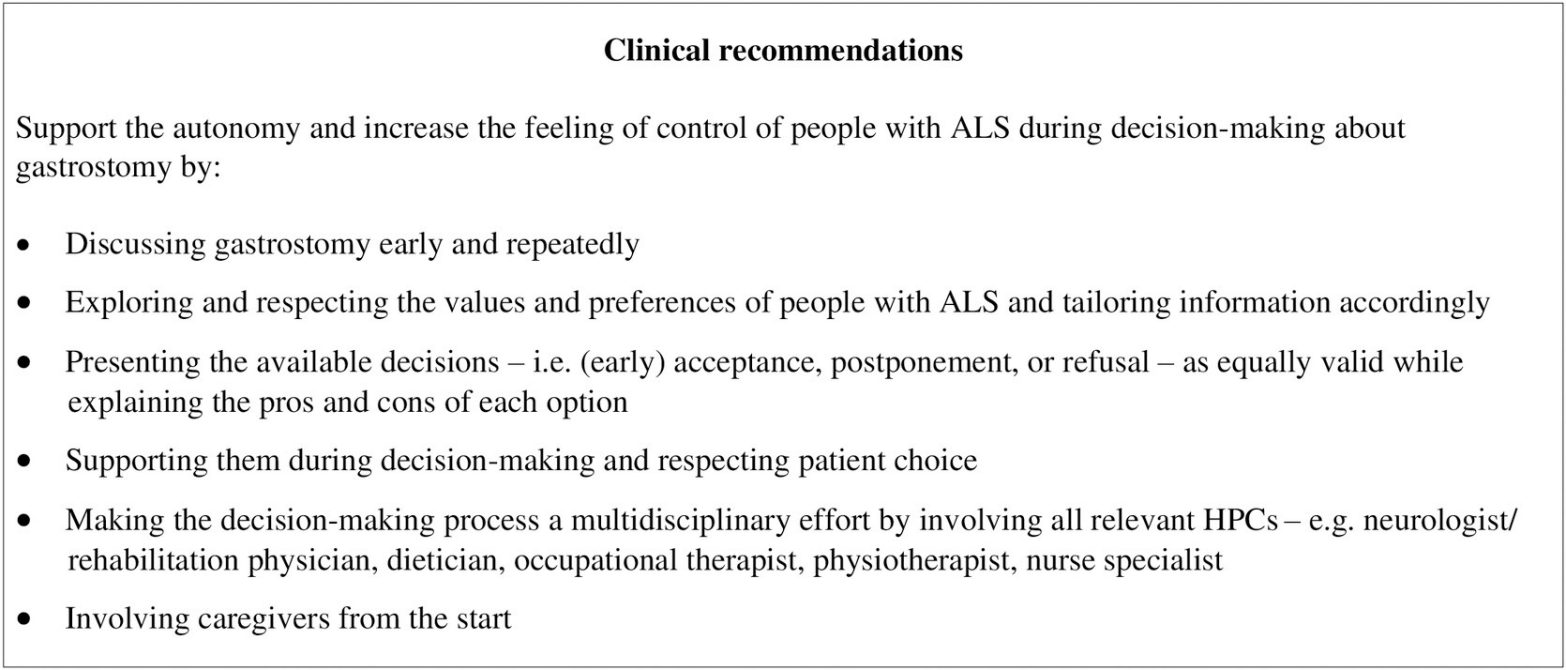
Clinical recommendations for decision-making about gastrostomy in ALS.

Person-centered decision-making on gastrostomy requires early information exchange, and repeated discussions by HCPs with people with ALS and their caregivers, in which their values are incorporated, and patient choice – i.e. accept, decline, or postpone gastrostomy – respected. This helps support the autonomy of people with ALS, makes them feel in control, and increases their satisfaction with the decision-making process (Fig 2).

## Supporting information

S1 File Interview guide patient and caregiver. (DOCX)

S2 File Interview guide with healthcare professionals. (DOCX)

S3 File COREQ checklist. (DOCX)

S4 File Researcher credentials. (DOCX)
