# Supplementary material for: Control in the absence of choice: A qualitative study on decision-making about gastrostomy in people with amyotrophic lateral sclerosis, caregivers, and healthcare professionals
Source: PLoS One. 2023 Sep 8;18(9):e0290508. doi: 10.1371/journal.pone.0290508 (PMC10490981; doi:10.1371/journal.pone.0290508)
Supplement: S1 File — (DOCX) [file pone.0290508.s001.docx]

**S1 File. Interview guide patient and caregiver: Decision-making about and experiences with gastrostomy in amyotrophic lateral sclerosis**

Interview guide with prompts.

**Personal information:**

Patient

Male/Female

Age:

Highest education:

Date diagnosis:

Type of feeding tube: PEG/PRG/other

Method of communication: verbal/non-verbal/verbal with communication aid

Caregiver

Male/Female

Age:

Highest education:

Relationship to patient:

**Interview**

1. To start off I would like to discuss the first time the topic of gastrostomy was first discussed with you. Can you remember this?
   1. Timing
   2. Response
   3. Did you have a notion what a feeding tube was?
2. I would also like to discuss your reasoning in deciding to accept or decline a feeding tube. Can you tell me how you came to this decision?
   1. Pros and cons
   2. Whose decision was it?
   3. Did you feel like you had a choice?
   4. Feelings surrounding the decision
   5. Differences between patient and caregiver
3. Can you tell me how the decision-making process proceeded and what you thought about it?
   1. Timing
   2. Information provision
   3. Role HCPs
   4. Role partner/family/caregiver
   5. Satisfaction with decision-making process
4. Looking back at the whole process, are there things you would have liked to do differently?
   1. Any regrets

**If the feeding tube was accepted:**

1. You chose to accept the feeding tube. I would like to ask you some follow-up questions about this. What are your experiences with the pros and cons of having a feeding tube?
   1. How do the pros compare to the cons?
   2. Did you expect this?
   3. Were you informed about this?
   4. What about the caregiver burden?

**[The following questions are to be asked in private due to their sensitive nature]**

**Questions to be asked of the caregiver in private:**

Introduction: Studies show that patients and their caregivers, partner or family sometimes have different opinions concerning the usefulness of interventions like the feeding tube. Some topics can also be sensitive. For these reasons I would like to ask you a few questions in private.

1. Mealtimes can be a heavy burden for caregivers. How did you experience mealtimes?
2. [if the feeding tube has been accepted] Did the burden of care surrounding mealtimes and feeding increase or decrease for you due to the placement of the feeding tube?
3. Cognitive changes in behavior or personality can occur during ALS and make the decision-making process more difficult. According to your experiences, have these kind of changes impacted the decision-making process about gastrostomy?

**Questions to be asked of the patient in private:**

Introduction: Studies show that patients and their caregivers, partner or family sometimes have different opinions concerning the usefulness of interventions like the feeding tube. Some topics can also be sensitive. For these reasons I would like to ask you a few questions in private.

1. For some patients caregiver burden can play a role in decision-making. Did this play a role for you in decision-making about gastrostomy?
2. Sometimes patients choose to accept interventions like a feeding tube due to pressure from their surroundings and not because it is their own decision. Did this play a role in your decision?
