# Supplementary material for: Control in the absence of choice: A qualitative study on decision-making about gastrostomy in people with amyotrophic lateral sclerosis, caregivers, and healthcare professionals
Source: PLoS One. 2023 Sep 8;18(9):e0290508. doi: 10.1371/journal.pone.0290508 (PMC10490981; doi:10.1371/journal.pone.0290508)
Supplement: S2 File — (DOCX) [file pone.0290508.s002.docx]

**S2 File. Interview guide healthcare professionals**

Interview guide with prompts on decision-making about and experiences with gastrostomy in amyotrophic lateral sclerosis.

**Personal information:**

Age:

Years of experience with ALS:

Type of feeding tube patient: PEG/PRG/other

**Interview**

Introduction: During this interview I would like to discuss the decision-making process about gastrostomy concerning one specific patient [name patient].

1. To start off, can you tell me when and how you first discussed the topic of gastrostomy with this patient?
   1. Response patient and caregiver
   2. Timing discussion
2. I would to dive deeper into the decision-making process about gastrostomy. Can you tell me more about this?
   1. Timing
   2. Arguments HCP (for and against)
   3. Response patient/caregiver
   4. Arguments patient/caregiver (for and against)
   5. Coming to a decision
3. What roles did the different participants take in the decision-making process?
   1. Who made the decision?
   2. Choice (yes/no)
   3. Role and relationship of patient and caregiver
   4. Possible cognitive impairments and their impact
4. Adequate provision of information is crucial to allow patients to make a well-informed decision about a feeding tube. According to you, what role did the information provision play in the decision-making process?
   1. Quality of the information
   2. Acceptance of need of gastrostomy (patient and caregiver)
5. What was the final decision?
   1. Timing placement (early, late, too late)
   2. Deciding factor
   3. Satisfaction with decision-making process
