# Supplementary material for: Control in the absence of choice: A qualitative study on decision-making about gastrostomy in people with amyotrophic lateral sclerosis, caregivers, and healthcare professionals
Source: PLoS One. 2023 Sep 8;18(9):e0290508. doi: 10.1371/journal.pone.0290508 (PMC10490981; doi:10.1371/journal.pone.0290508)
Supplement: S3 File — (DOCX) [file pone.0290508.s003.docx]

**S3 Table. COREQ checklist**

| **Topic** | **Item No.** | **Guide questions/ description** | **Answer** |
| --- | --- | --- | --- |
| **Domain 1: Research team and reflexivity** | | | |
| *Personal characteristics* | | | |
| Interviewer/facilitator | 1 | Which author/s conducted the interview or focus group? | Remko van Eenennaam and Neele Rave, see Methods – Data collection. |
| Credentials | 2 | What were the researcher’s credentials? E.g. PhD, MD | See S4 Table. |
| Occupation | 3 | What was their occupation at the time of the study? | See S4 Table. |
| Gender | 4 | Was the researcher male or female? | Not relevant for this study. |
| Experience and training | 5 | What experience or training did the researcher have? | See Methods – Data collection. |
| *Relationship with participants* | | | |
| Relationship established | 6 | Was a relationship established prior to study commencement? | Participants were contacted by phone prior to the interview and informed about the study. The role of the interviewers was explained to participants. Other than that the interviewers were unknown to patients and caregivers.  See Methods – Participants. |
| Participant knowledge of the interviewer | 7 | What did the participants know about the researcher? e.g. personal goals, reasons for doing the research | As far as the patients and caregivers were concerned, no background knowledge of the interviewers was known to the participants, except for their role in the study. The background of the study was explained to participants.  One interviewer (RvE) was known to two of the HCPs prior to them being interviewed.  See Methods – Participants. |
| Interviewer characteristics | 8 | What characteristics were reported about the interviewer/facilitator? e.g. Bias, assumptions, reasons and interests in the research topic | Neither interviewer was involved in patient care.  See Methods – Participants and S4 Table. |
| **Domain 2: Study design** | | | |
| *Theoretical framework* | | | |
| Methodological orientation and Theory | 9 | What methodological orientation was stated to underpin the study? e.g. grounded theory, discourse analysis, ethnography, phenomenology, content analysis | Interviews were analyzed using an inductive approach and categorized into overarching (sub)themes using thematic analysis.  See Methods – Data analysis. |
| *Participant selection* | | | |
| Sampling | 10 | How were participants selected? e.g. purposive, convenience, consecutive, snowball | See Methods - Participants. |
| Method of approach | 11 | How were participants approached? e.g. face-to-face, telephone, mail, email | See Methods - Participants. |
| Sample size | 12 | How many participants were in the study? | See Results – Participants and Table 1. |
| Non-participation | 13 | How many people refused to participate or dropped out? Reasons? | None. |
| *Setting* | | | |
| Setting of data collection | 14 | Where was the data collected? e.g. home, clinic, workplace | Data was collected electronically via email, and telephone or video-consultation.  See Methods – Data collection. |
| Presence of nonparticipants | 15 | Was anyone else present besides the participants and researchers? | During the interviews no one else was present except for the participants and the interviewers. |
| Description of sample | *16* | What are the important characteristics of the sample? e.g. demographic data, date | See Table 1. |
| *Data collection* | | | |
| Interview guide | 17 | Were questions, prompts, guides provided by the authors? Was it pilot tested? | The interview guides can be found in S1 File and S2 File. The interview guide was not field tested. |
| Repeat interviews | 18 | Were repeat interviews carried out? If yes, how many? | No repeat interviews were conducted. |
| Audio/visual recording | 19 | Did the research use audio or visual recording to collect the data? | Yes, see Methods - Participants. |
| Field notes | 20 | Were field notes made during and/or after the interview or focus group? | Field notes were made during the interviews to support the interviewer. These were not analysed or recorded after the interview. |
| Duration | 21 | What was the duration of the inter views or focus group? | See Results – Participants. |
| Data saturation | 22 | Was data saturation discussed? | Yes, see Methods – Data analysis |
| Transcripts returned | 23 | Were transcripts returned to participants for comment and/or corrected? | Yes, see Methods – Data collection. |
| **Domain 3: analysis and findings** | | | |
| *Data analysis* | | | |
| Number of data coders | 24 | How many data coders coded the data? | Two (RvE and NR).  See Methods – Data analysis. |
| Description of the coding tree | 25 | Did authors provide a description of the coding tree? | The coding tree is available (in Dutch) at request from the corresponding author. |
| Derivation of themes | 26 | Were themes identified in advance or derived from the data? | Themes were derived from the data.  See Methods – Data analysis. |
| Software | 27 | What software, if applicable, was used to manage the data? | NVIVO 12.  See Methods – Data analysis. |
| Participant checking | 28 | Did participants provide feedback on the findings? | No. |
| *Reporting* | | | |
| Quotations presented | 29 | Were participant quotations presented to illustrate the themes/findings? Was each quotation identified? e.g. participant number | Yes, see tables 2-5. |
| Data and findings consistent | 30 | Was there consistency between the data presented and the findings? | Yes, see Tables 2-5 and Results section. |
| Clarity of major themes | 31 | Were major themes clearly presented in the findings? | Yes, see Tables 2-5 and Results section. |
| Clarity of minor themes | 32 | Is there a description of diverse cases or discussion of minor themes? | Yes, see Tables 2-5 and Results section. |
