# Supplementary material for: Control in the absence of choice: A qualitative study on decision-making about gastrostomy in people with amyotrophic lateral sclerosis, caregivers, and healthcare professionals
Source: PLoS One. 2023 Sep 8;18(9):e0290508. doi: 10.1371/journal.pone.0290508 (PMC10490981; doi:10.1371/journal.pone.0290508)
Supplement: S4 File — (DOCX) [file pone.0290508.s004.docx]

**S4 Table. Researcher credentials**

| **Name** | **Occupation** |
| --- | --- |
| Remko M. van Eenennaam, MSc | Researcher (PhD-student) |
| Neele Rave, MSc | Researcher (PhD-student) |
| Willeke Kruithof, MD, PhD | Rehabilitation physician |
| Esther Kruitwagen-van Reenen, MD, PhD | Rehabilitation physician |
| Leonard H. van den Berg, MD, PhD | Neurologist |
| Anne Visser-Meily, MD, PhD | Rehabilitation physician |
| Anita Beelen, PhD | Senior researcher |
